# 

**Published:** 1889-06

**Authors:** 


					﻿io cts. a copy
JUNE, 1883.
$i.oo per year.
HALLS®
gJOVRNAW
HEALTH
ESTABLISHED 1854
U°±- 36
No. 6
OFFICE : 206 BROADWAY, EVENING POST BUILDING, Boom 11. N. Y.
Entered as Second-class Matter at the Post-Office, New York.
				

